# Hsa-microRNA-101 suppresses migration and invasion by targeting Rac1 in thyroid cancer cells

**DOI:** 10.3892/ol.2014.2361

**Published:** 2014-07-18

**Authors:** CHENGHAI WANG, SIJIA LU, JIXIN JIANG, XIAOQIN JIA, XIAOYUN DONG, PING BU

**Affiliations:** 1 Department of Pathology, Medical College of Yangzhou University, Yangzhou, Jiangsu 225001, P.R. China; 2 Department of Pathology, The Affiliated Jiangsu Subei Hospital, Yangzhou University, Yangzhou, Jiangsu 225001, P.R. China; 3 Department of Chinese and Western Integrative Medicine, Medical College of Yangzhou University, Yangzhou, Jiangsu 225001, P.R. China

**Keywords:** adenocarcinoma, biopsy, thyroid cancer, Rac1, miR-101, migration, invasion

## Abstract

MicroRNAs (miRNAs) are 22- to 25-nucleotide non-coding RNA molecules that function as negative regulators of gene expression. In previous years, increasing evidence has arisen implicating the involvement of miRNAs in carcinogenesis. In previous studies, the role of miRNA-101 (miR-101) in tumors has been identified as a tumor suppressor and, until now, the role of miR-101 and Rac1 in thyroid cancer has remained undefined. This study revealed that miR-101 is significantly downregulated in papillary thyroid carcinoma (PTC) tissue and thyroid cancer cell lines, and that the downregulated miR-101 is associated with lymph node metastasis. Infection with the miR-101 murine stem cell virus may markedly inhibit cell migration and invasion in TPC-1 and HTH83 thyroid cancer cell lines. Rac1 was demonstrated to be negatively regulated by miR-101 at the post-transcriptional level, via a specific target site within the 3′ untranslated region by dual-luciferase reporter assay. The expression of Rac1 was also observed to inversely correlate with miR-101 expression in PTC tissues; knockdown of Rac1 by shRNA inhibited thyroid cancer cell migration and invasion, resembling that of miR-101 overexpression. Thus, these findings suggested that miR-101 acts as a novel suppressor by targeting the Rac1 gene and inhibiting thyroid cancer cell migration and invasion.

## Introduction

Thyroid carcinoma is the most common type of primary endocrine malignancy of the thyroid in adults. The estimated worldwide incidence rate is ~1.7% of total cancer diagnoses (1), and the incidence has increased over the last decades. The mortality rate of thyroid carcinoma has remained stable for a number of years, with a rate of 0.368 per 100,000 individuals in China (2). Thyroid nodules are diagnosed in >5% of the adult population and may be benign adenomas or malignant lesions. There are four types of thyroid cancer: Papillary, follicular, medullary and anaplastic. Papillary cancer is the most common type of thyroid cancer, accounting for 80–90% of all cases. Papillary thyroid carcinoma (PTC) is extremely easy to treat and, in numerous cases, curable. While papillary thyroid tumors often spread to the cervical lymph nodes (3), they do not commonly spread to distant organs, as observed in other types of cancer. If metastasis occurs, it frequently involves the bones and lungs. The metastasis of thyroid carcinoma is the major cause of fatal outcome and, therefore, it is essential to identify metastasis-associated molecules and to gain an improved understanding of the mechanisms involved in the metastasis of thyroid carcinoma (4,5).

MicroRNAs (miRNAs) are 22- to 25-nucleotide non-coding RNA molecules that typically prohibit protein production by binding to the 3′ untranslated region (UTR) of specific target mRNAs (6–8). This mutual effect recruits the RNA-induced silencing complex (RISC) that facilitates nucleolytic cleavage and/or inhibits the translation of target mRNAs. By regulating protein yield post-transcriptionally, miRNAs transform the formation and function of all tissues. Furthermore, certain miRNAs may serve as oncogenes or tumor suppressor genes in the development of tumors. However, the regulation of the majority of miRNAs and their precise mechanisms of action remain unknown for thyroid carcinoma.

miRNA-101 (miR-101) is a tumor suppressor miRNA. Existing studies have indicated that miR-101 is markedly downregulated in a number of carcinomas, including those of the stomach, liver, breast, prostate and endometrium. Furthermore, miRNA has been shown to effect tumor cell proliferation, migration and invasion (9–14). However, little is known with regard to the expression level and biological role of miR-101 in PTC.

In the current study, the differential expression of miR-101 in human PTC samples was identified using quantitative polymerase chain reaction (qPCR), and the function of miR-101 in migration and invasion of thyroid cancer cells was investigated. In addition, to understand the molecular mechanism of thyroid cancer metastasis, the target gene of miR-101 was further investigated. To the best of our knowledge, this is the first study to investigate the expression and mechanism of miR-101 in thyroid carcinoma migration and invasion. This study presents a novel target for further therapeutic studies of thyroid cancer.

## Materials and methods

### PTC tissue collection

A total of 16 paired tissue specimens from PTC and matched normal tissue were obtained from 16 PTC patients (age range, 40–62 years; nine males and seven females) at the Departments of Surgery of the Jiangsu Subai and Yangzhou Chinese Medical Hospitals, affiliated to Yangzhou University (Yangzhou, China). The absence of tumor cells in the matched normal tissues was confirmed by a pathologist. All tissues were obtained during surgery and immediately stored in liquid nitrogen prior to use. Approval for this study was granted by the Institute Research Medical Ethics Committee of the Medical College of Yangzhou University (Yangzhou, China). Patients provided written informed consent.

### Cell line culture

TPC-1 cells were provided by Dr Sissy Jhiang (Ohio State University, Columbus, OH, USA) and HTH83 cells were provided by Dr Nils-Erik Heldin (University Hospital, Uppsala, Uppsala, Sweden). TPC-1, HTH83 and 293T cells (Fengshou, Shanghai, China) were cultured in Dulbecco’s modified Eagle’s medium, containing 10% fetal bovine serum (FBS; HyClone, Victoria, Australia), 100 IU/ml penicillin and 100 mg/ml streptomycin (Beyotime Institute of Biotechnology, Haimen, China), at 37°C in a 5% CO_2_ humidified atmosphere.

### qPCR

Total RNAs were isolated from cells using TRIzol reagent (Invitrogen Life Technologies, San Diego, CA, USA) and reverse transcription was performed using the PrimeScript™ First Strand cDNA synthesis kit (Takara Bio, Inc., Dalian, Japan) according to the manufacturer’s instructions. qPCR was performed using the All-in-One™ miRNA qRT-PCR Detection Kit (GeneCopoeia, Rockville, MD, USA) on an Applied Biosystems 7500 Real-Time PCR system (Applied Biosystems, White Plains, NY, USA). The U6 small RNA and β-actin mRNA were used as internal controls. All the reactions were run in triplicate and the following primers were used: Forward, 5′-GAAGATCTCCAGCCACCTGTTTCACA-3′ and reverse, 5′-CCGCTCGAGTAGTCCTTCACTTCATG-3′ for miR-101; forward, 5′-CGCTTCGGCAGCACATATAC-3′ and reverse, 5′-TTCACGAATTTGCGTGTCAT-3′ for U6; forward, 5′-AGGAAGGCGGACATATTAGTCCCT-3′ and 5′-AGACGATAGTTGGGTCCCGGC-3′ for Rac1; and forward, 5′-GTCACCAACTGGGACGACAT-3′ and reverse, 5′-GAGGCGTACAGGGATAGCAC-3′ for β-actin mRNA.

### Production of retroviral particles and infection of thyroid cancer cells

Rac1 shRNA was purchased from Santa Cruz Biotechnology, Inc. (Santa Cruz, CA, USA). The miR-101-murine stem cell virus (MSCV) plasmid and pRac-1-shRNA-MSCV were chemically synthesized at the Department of Pathology, Medical College of Yangzhou University (Yangzhou, Jiangshu) and sequenced by Sangon Biotech (Shanghai) Co., Ltd. (Shanghai, China). 293T cells were seeded on 90-mm dishes one day prior to transfection. Next, 10 μg of retroviral plasmids together with 15 μg of miR101-MSCV vector were used to cotransfect 293T cells by Lipofectamine 2000 (Invitrogen Life Technologies), according to the manufacturer’s instructions. The retroviral supernatants were collected 48 h following transfection and stored at −80°C. Following this, 5–8 ml of supernatant containing the miR-101-MSCV virus together with 8 μg/ml polybrene (Sigma-Aldrich, New York, NY, USA) were used to infect the TPC-1 and HTH83 cells. For the infection of thyroid cancer cells, the cells were incubated at 37°C in a 5% CO_2_ humidified atmosphere (15). TPC-1 and HTH83 cells were also infected with shRac1-MSCV virus or MSCV empty vector as negative control (NC). The miR-101 and Rac1 RNA levels in the infected thyroid cancer cells were identified by qPCR.

### Dual-luciferase reporter assay

The full-length 3′-UTR of Rac1 was amplified by PCR from genomic DNA and cloned into the *Eco*RI and *Xha*I sites of the pGL3-BS vector (Promega Corporation, Madison, WI, USA). The primer sequences used were as follows: Forward, 5′-GTGAATTCACTGGTTGTTCTGTTAGTCGCT-3′ and reverse, 5′-GTTCTAGACCAGTCGTATGATTCAAGGATTT-3′ for Rac1 3′-UTR. The mutant construct of Rac1 3′UTR was generated using a Quick Change mutagenesis kit (Stratagene, Heidelberg, Germany). Cotransfection of the reporter vectors, and miR-101 mimics or NCs was performed using Lipofectamine 2000 (Invitrogen Life Technologies). After 48 h, dual-luciferase activity was measured using the Dual-Luciferase^®^ reporter assay system (Promega Corporation) according to the manufacturer’s instructions.

### Migration and invasion assay

*In vitro* cell migration and invasion assays were performed using Transwell chambers. For the migration assays, 5×10^4^ cells were added to the upper chamber of 8-μm pore size Transwells (BD Biosciences, Franklin Lakes, NJ, USA). For the invasion assays, 1×10^5^ cells were added to the upper chamber of 8-μm pore size Tranwells precoated with Matrigel (BD Biosciences). In these assays, cells were plated in medium without serum and medium containing 10% FBS in the lower chamber, serving as a chemoattractant. After 14 h of incubation, the cells that had not migrated or invaded through the pores were carefully removed. The filters were then fixed in 90% alcohol, which was followed by crystal violet staining. Five random fields were counted per chamber using an inverted microscope (CKX41; Olympus Corporation, Tokyo, Japan) and each test was performed in triplicate.

### Western blot analysis

Proteins were extracted using cell lysis buffer for western and immunoprecipitation (Beyotime Institute of Biotechnology) according to the manufacturer’s instructions. The protein concentration was quantified using the Enhanced BCA Protein Assay kit (Beyotime Institute of Biotechnology). For western blot analysis, equal amounts of total protein were boiled and separated by SDS-PAGE. Following electrophoresis, the protein was blotted onto a polyvinylidene fluoride membrane and blocked for 2 h at room temperature. The membranes were then incubated with human monoclonal anti-rabbit Rac1 antibody (Cell Signaling Technologies, Boston, MA, USA) at 1:1,000 dilutions overnight at 4°C. The Rac1 protein level was then detected by goat polyclonal anti-mouse horseradish peroxidase-conjugated secondary antibodies (Beyotime Institute of Biotechnology) for 2 h at room temperature. The protein bands were detected using a FluorChem FC2 imaging system (Alpha Innotech, San Leandro, CA, USA).

### Statistical analysis

Statistical analyses were performed using SPSS 16.0 (SPSS Inc., Chicago, IL, USA). All graphs were created using Microsoft Office Excel 2010 software (Microsoft Corporation, Redmond, WA, USA). All data from three independent experiments are presented as the mean ± standard deviation. The differences were assessed by two-tailed Student’s t-test, while the correlation between RAC1 and miR-101 expression was investigated using the two-tailed Pearson’s correlation. P<0.05 was considered to indicate a statistically significant difference.

## Results

### Expression of miR-101 is downregulated in PTC tissues and cell lines

**Figure 1:**
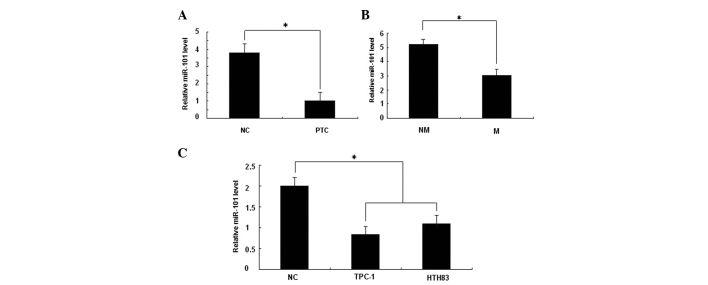
Hsa-miR-101 is downregulated in PTC. (A) Relative expression levels of miR-101 were determined by quantitative polymerase chain reaction in 16 paired human PTC and their corresponding normal samples, which were normalized against an endogenous U6 RNA control. Student’s t-test was used to analyze the significant differences between the tumor and normal tissues. (B) Relative expression levels of miR-101 in PTC with lymph node metastasis (M; n=11) and non-metastasis (NM; n=5). Student’s t-test was used to analyze the significant differences. (C) Relative expression levels of miR-101 in the two thyroid cancer cell lines and human thyroid tissue cells. Data are presented as the mean ± standard deviation. Two-tailed Student’s t-test was used to analyze the significant differences. ^*^P<0.05. miR, microRNA; PTC, papillary thyroid tumor; NC, negative control.

Due to the downregulation of miR-101 in human melanoma, the downregulation of miR-101 in human thyroid tumors was investigated for comparison. The endogenous expression of miR-101 in human PTC and the adjacent normal thyroid tissues was compared by qPCR. As shown in Fig. 1A, the expression of miR-101 was downregulated in 93.75% (15 out of 16) of PTC tissues, compared with the corresponding adjacent normal thyroid tissues. The expression of miR-101 was also observed to be further downregulated in 68.75% (11 out of 16) of PTCs with lymph node metastasis, when compared with those without lymph node metastasis (P<0.05; Fig. 1B). Similarly, a decrease in the expression of miR-101 was observed in two thyroid cancer cell lines, compared with the human thyroid tissue cells (Fig. 1C). Collectively, the above findings suggested that the loss of miR-101 expression may be significant in PTC development and metastasis.

### Overexpression of miR-101 inhibits thyroid cancer cell migration and invasion

**Figure 2:**
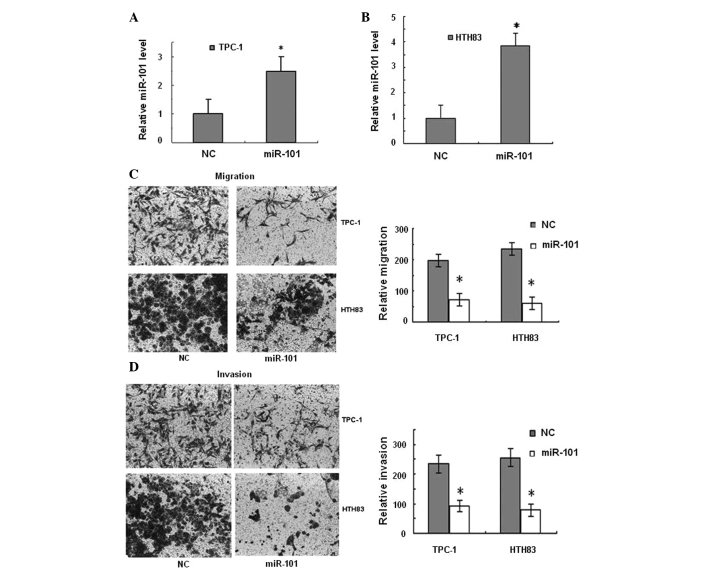
Overexpression of miR-101 inhibits the migration and invasion of thyroid cancer cell lines. Infection of miR-101-murine stem cell virus to (A) TPC-1 and (B) HTH83 increased the expression of miR-101 as detected by quantitative polymerase chain reaction. The inhibitory effect of miR-101 toward the (C) migration and (D) invasion of TPC-1 and HTH83 cells. Data are presented the as mean ± standard deviation. Two-tailed Student’s t-test was used to analyze the significant differences. ^*^P<0.05, vs. the corresponding NC. miR, microRNA; NC, negative control.

Based on the aforementioned results, the possibility that miR-101 is involved in modulating the migration and invasion of thyroid cancer cells was investigated. TPC-1 and HTH83 cells were infected with miR-101-MSCV or the NC and then evaluated by cell invasion and migration assays. As predicted, infection with miR-101-MSCV increased miR-101 expression compared with the NC in TPC-1 and HTH83 cells (Fig. 2A). Furthermore, the cell migration and invasion assays indicated that miR-101 overexpression results in a reduced migration and invasion rate in TPC-1 and HTH83 cells compared with the control (Fig. 2B). The results also indicated that miR-101 acts as a tumor suppressor miRNA, and contributes to the inhibition, migration and invasion of thyroid cancer cells.

### miR-101 negatively regulates Rac1 gene expression

**Figure 3:**
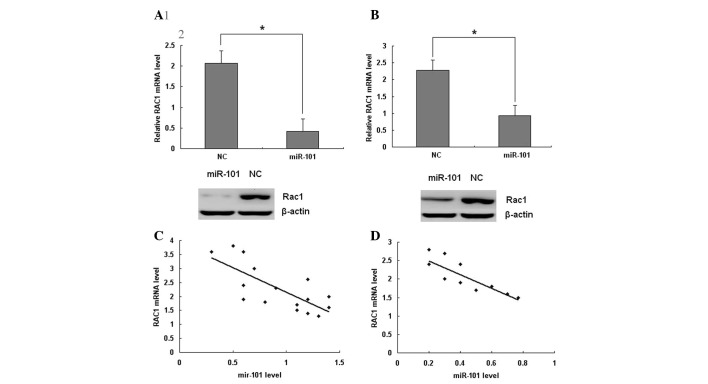
miR-101 negatively regulates Rac1 gene expression. miR-101 inhibited the expression of Rac1 at the mRNA and protein level in thyroid cancer (A) cells and (B) tissues. Data are presented as the mean ± standard deviation. Two-tailed Student’s t test was used to analyze the significant differences. ^*^P<0.05, vs. the NC. Analysis of the correlation between miR-101 and Rac1 expression in (C) PTC tissues (two-tailed Pearson’s correlation analysis; r=0.750; P<0.05) and (D) PTC tissues with lymph node metastasis (two-tailed Pearson’s correlation analysis; r=0.820; P<0.05) by detecting the mRNA expression. miR, microRNA; PTC, papillary thyroid tumor; NC, negative control.

miRNAs are known to suppress hundreds of mRNA targets, resulting in global changes in the cellular phenotype of cells (16). Initially, potential targets for miR-101 were identified using the prediction software, TargetScan (www.targetScan.org). The Rac1 gene was identified as the putative target gene for miR-101, which mediates cell migration and invasion. To further confirm that Rac1 was a target gene for miR-101, qPCR and western blot analysis were used to detect the expression of Rac1 regulated by miR-101 in TPC-1 and HTH83 cells. Following the overexpression of miR-101, the expression of Rac1 was significantly downregulated at the mRNA and protein level (Fig. 3A and 3B) when compared with the NC. Furthermore, the mRNA levels of miR-101 and Rac1 were also detected in PTC and the adjacent normal thyroid tissues by qPCR. The inverse correlation between miR-101 and Rac1 in PTC was further investigated, and the Rac1 mRNA and miR-101 expression levels were determined in the same PTC specimens by qPCR. As shown in Fig. 3C, when the Rac1 mRNA levels were plotted against miR-101 expression, a significant inverse correlation was observed (two-tailed Pearson’s correlation analysis; r=−0.750; P<0.05). Similarly, as shown in Fig. 3D, a significant inverse correlation was also observed for PTC specimens with lymph node metastasis (two-tailed Pearson’s correlation analysis; r=−0.820; P<0.05). Overall, these results suggested that miR-101 negatively regulates the Rac1 gene expression at the transcription level, and that Rac1 is a potential target gene of miR-101.

### Rac1 is a direct target of miR-101

**Figure 4:**
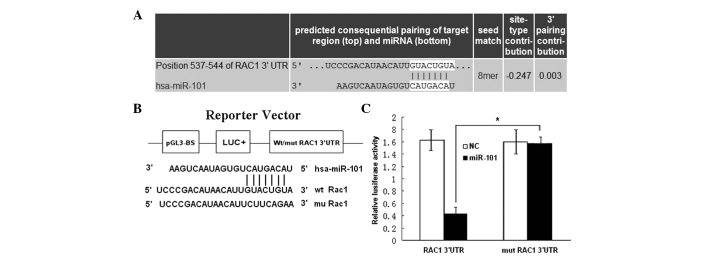
Rac1 3′UTR is a target of miR-101. (A) Rac1 was the predicted target gene of miR-101 as determined by microRNA.org. (B) Diagram of the luciferase reporter plasmids with the wild-type or mutant Rac1 3′UTR. (C) The relative luciferase activity in 293T cells was determined after the plasmids with wild-type or mutant Rac1 3′UTR had been cotransfected with miR-101 mimics. Data are presented as the mean ± standard deviation. Two-tailed Student’s t-test was used to analyze the significant differences. ^*^P<0.05. UTR, untranslated region; miR, microRNA; LUC, luciferase.

To verify whether miR-101 directly targets Rac1, dual-luciferase reporter assays were conducted. As shown in Fig. 4, cotransfection of 293T cells with Rac1-3′UTR/pGL3-BS and miR-101 mimics caused a significant decrease in the luciferase activity compared with the NC (P<0.05). This repressive effect was eliminated by introducing point mutations into the core binding sites of the Rac1 3′-UTR. This result indicated that miR-101 exerts an inhibitory effect on Rac1 expression via interactions with the 3′UTR of Rac1.

### Knockdown of Rac1 reduces the migration and invasion potential of thyroid cancer cells

**Figure 5:**
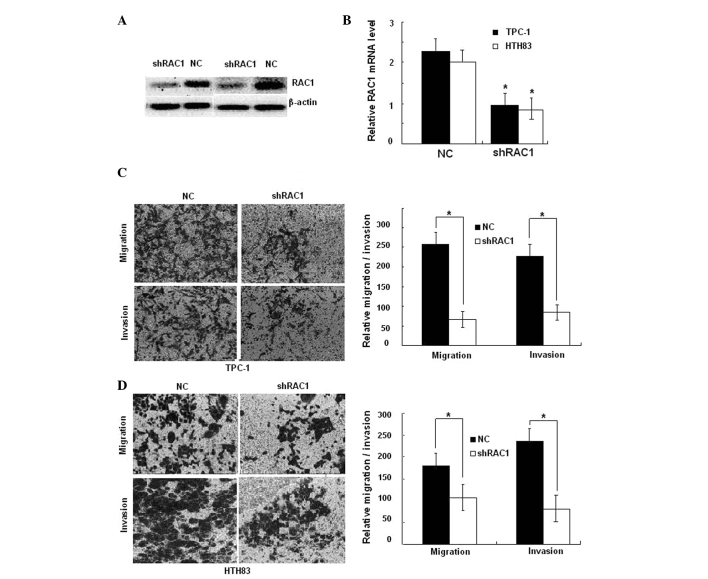
Knockdown of Rac1 reduces the migration and invasion in TPC-1 and HTH83 cells. Infection of Rac1 shRNA-murine stem cell virus inhibited the (A) protein and (B) mRNA expression of Rac1 in TPC-1 and HTH83 cells. Knockdown of Rac1 by shRNA inhibited migration and invasion in (C) TPC-1 and (D) HTH83 cells. Data are presented as the mean ± standard deviation. Two-tailed Student’s t-test was used to analyze the significant differences. ^*^P<0.05, vs. the corresponding NC. miR, microRNA; NC, negative control.

To confirm the effects of Rac1 on the migration and invasion of PTC cells, Rac1 expression was knocked down by a Rac1 shRNA. The mRNA and protein expression of Rac1 was significantly downregulated in TPC-1 and HTH83 cells following Rac1 shRNA treatment (Fig. 5A and B). Consistently, the knockdown of Rac1 significantly reduced cell migration and invasion in TPC-1 and HTH83 cells, which resembled the inhibitory effects of miR-101 (Fig. 5C and D). These results suggested that the knockdown of Rac1 suppresses the migration and invasion of PTC cells and that Rac1 is an effective target gene of miR-101.

## Discussion

miRNAs are 22- to 25-nucleotide non-coding RNA molecules that typically prohibit protein production by binding to the 3′UTR of specific target mRNAs. This mutual effect recruits RISC, which facilitates nucleolytic cleavage or inhibits translation of target mRNAs. By regulating the protein production post-transcriptionally, a number of miRNAs act as oncogenes or tumor suppressor genes. However, the regulation of miR-101 and its precise mechanisms of action in thyroid cancers remain unknown.

The results presented in this study indicated that miR-101 exhibits lower expression in PTC tissues compared with the matched normal thyroid tissues. miR-101 was also observed to inhibit the invasion and migration of two thyroid cancer cell lines. Overall, the results suggested that miR-101, as a newly identified tumor suppressor, is involved in the metastasis and infiltration of PTC. Furthermore, Rac1 was identified as a potential target of miR-101 by theoretical prediction. Rac1 is one of the most studied Rho GTPase proteins (17). It contributes to cell proliferation, participates in the signaling pathway promoting cell survival, and is known for its central role in the control of cell adhesion and migration (18,19). It has also been reported that Rac1 is overexpressed in colorectal and lung tumors (20,21), and is associated with metastasis and invasion in breast, upper urinary tract and oral squamous cell tumors (22–25).

Three pathways have been associated with the molecular mechanism of the knockdown of Rac1-inhibited migration and invasion: i) Downregulation of Rac1 targets miR-124, leading to inactivation of the MKK4/JNK/c-Jun pathway (26); ii) loss of miR-204-targeting Rac1 results in activation of brain-derived neurotrophic factor and subsequent activation of Rac1 through the AKT/mTOR signaling pathway, leading to cancer cell migration and invasion (27); and iii) downregulation of diallyl disulfide-suppresses SW480 cell migration and invasion through the Rac1-ROCK1/PAK1-LIMK1-ADF/cofilin signaling pathway (28).

In the current study, an inverse correlation was observed between the miR-101 and Rac1 expression in PTC tissues. The results also demonstrated that Rac1 is negatively regulated by miR-101 at the post-transcriptional level via a specific target site within the 3′UTR, and that miR-101 inhibits thyroid cell migration and invasion through the Rac1 pathway. Overall, these results suggested that elevated Rac1, induced by suppressed miR-101, is involved in the progression of PTC.

This evidence suggests that the dysregulation of the Rac1 signaling pathway by miRNAs is an important mechanism underlying cancer metastasis, particularly in cancer cell migration and cell invasion.

Metastasis is the movement or spread of cancer cells between one organ or tissue to another. Its sequential events include detachment, migration, local invasion, formation of tumor emboli, extravasations and transplantation in various organs (29,30). Certain miRNAs may regulate the signaling pathways of tumor metastasis (31), and the identification of miR-101 as an important regulator of tumor cell migration and invasion *in vitro* emphasizes an essential role of this miRNA in mediating PTC oncogenesis and tumor behavior.

In conclusion, the results presented in the current study demonstrate that miR-101 is downregulated in PTC and that the downregulated miR-101 is significantly associated with lymph node metastasis. Furthermore, miR-101 inhibits the invasion and migration in thyroid cancer cells; miR-101 directly inhibited Rac1 expression by targeting its 3′UTR. In addition, Rac1 was downregulated and found to inversely correlate with miR-101 levels in thyroid carcinoma. These results suggest that miR-101 is an important tumor suppressor in thyroid carcinoma. This study may lead to the development of novel therapies for preventing thyroid cancer metastasis.
